# Instrumental indices for upper limb function assessment in stroke patients: a validation study

**DOI:** 10.1186/s12984-016-0163-4

**Published:** 2016-06-08

**Authors:** Maria Longhi, Andrea Merlo, Paolo Prati, Meris Giacobbi, Davide Mazzoli

**Affiliations:** Gait & Motion Analysis Laboratory, Sol et Salus Hospital, viale San Salvador 204, Rimini, 47922 Torre Pedrera di Rimini, Italy; Rehabilitation Department, Motion Analysis Laboratory, AUSL of Reggio Emilia, via Mandriolo Superiore 11, Correggio, 42015 Reggio Emilia, Italy

**Keywords:** Upper limb, Robotic indices, Validity, Stroke, Rehabilitation

## Abstract

**Background:**

Robotic exoskeletons are increasingly being used in objective and quantitative assessment of upper limb (UL) movements. A set of instrumental indices computed during robot-assisted reaching tasks with the Armeo®Spring has been proven to assess UL functionality. The aim of this study was to test the construct validity of this indices-based UL assessment when used with patients who have had a stroke.

**Methods:**

Forty-four 45- to 79-year-old stroke patients with a Wolf Motor Function Test ability score (WMFT-FAS) ranging from 10 to 75 and a Motricity Index (MI) ranging from 14 to 33 at shoulder and elbow were enrolled, thus covering a wide range of impairments. Residual UL function was assessed by both the WMFT-FAS and the WMFT-TIME, as well as by a set of 9 numerical indices assessing movement accuracy, velocity and smoothness computed from a 3D endpoint trajectory obtained during the “Vertical Capture” task of the Armeo®Spring device. To explore which variables better represented motor control deficits, the Mann-Whitney *U* Test was used to compare patients’ indices to those obtained from 25 healthy individuals. To explore the inner relationships between indices and construct validity in assessing accuracy, velocity and smoothness, a factor analysis was carried out. To verify the indices concurrent validity, they were compared to both WMFT-FAS and WMFT-TIME by the Spearman’s correlation coefficient.

**Results:**

Seven indices of stroke subjects were significantly different from those of healthy controls, with effect sizes in the range 0.35–0.74. Factor analysis confirmed that specific subsets of indices belonged to the domains of accuracy, velocity and smoothness (discriminant validity). One accuracy index, both velocity indices and two smoothness indices were significantly correlated with WMFT-FAS and WMFT-TIME (|*rho*| = 0.31–0.50) (concurrent validity). One index for each of the assessed movement domains was proven to have construct validity (discriminant and concurrent) and was selected. Moreover, the indices were able to detect differences in accuracy, velocity and/or smoothness in patients with the same WMFT level.

**Conclusions:**

The proposed index-based UL assessment can be used to integrate and support clinical evaluation of UL function in stroke patients.

## Background

Upper-extremity impairment is the most significant and persistent physical disability in stroke survivors, being reported in about 70 % of patients on admission subsequent to a stroke [1]. This may determine a great reduction in the reaching ability needed to perform everyday activities and, consequently, in their independence [2]. Quantifying the reaching ability in stroke patients, which is necessary to determine the initial loss of movement and function, is essential in order to track the motor recovery throughout the rehabilitation period, and relies on clinical scales. The most common scoring systems in this field are the Fugl-Meyer Motor Performance Assessment (FMA) [3–6] to assess motor impairment and the WMFT [7–9] to evaluate UL function. With respect to the International Classification of Functioning, Disability and Health (ICF), [10] the FMA only deals with the body function domain, while the WMFT is classified in terms of ICF categories as an Activity Scale, as it measures changes in functional activities [11]. These tests have proven their reliability [12] and validity [13–15] when assessing UL function in stroke patients, despite some minor limitations [16–18], such as equipment needs, time of execution, training requirements and the lack of sensitivity to subtle changes in motor performance throughout the rehabilitation process.

The recovery in UL movement, if any, typically begins in the sub-acute stage after a stroke and is supported by personalized rehabilitation programs, the positive outcome of which is known to depend on repetition, intensity and task-orientation [19]. For this reason, robotic devices and exoskeletons that support the upper limb during execution of repetitive tasks have been introduced as part of overall treatment aimed at restoring upper extremity functionality [20–22]. Apart from their use in the rehabilitative practice, the availability of embedded sensors could permit a further use as an assessment device. In fact, UL robotic rehabilitation devices have recently been used as an objective and accurate assessment tool to monitor progress [23] and to collect quantitative data that summarize individual performance [24]. The duration of robot-based tests, as reported in related literature, lasts generally no less than 30–40 minutes [25–27], with the number of parameters extracted from the robot-assisted upper limb trajectory being largely variable among studies, varying from 5 smoothness parameters in the study by Roher and colleagues [28] up to 22 indices that contributed to creating a single composite score in the paper by Einav et al. [25]. In general, as summarized in the review by Nordin et al. [29], several indicators have been proposed in the literature to describe UL impairment in stroke patients, dealing with movement planning, accuracy, coordination, efficiency, velocity, acceleration, jerkiness and number of movement sub-units. However, a complete analysis of their metric characteristics and concurrent validity with clinical scales is still missing [29].

We have recently developed a customized stand-alone software that increases the usefulness of a commercially available exoskeleton (Armeo®Spring, Hocoma AG, Switzerland), by providing a set of indices aimed at assessing accuracy, velocity and smoothness of UL motion along with the related reference values [30]. When used with stroke patients, this assessment can be carried out between 5 to 15 min, depending on the patient’s impairment level. The derived indices are easy to share with clinical professionals, as they refer directly to movement accuracy, velocity and lack of smoothness (jerkiness), which are clinical aspects of UL impairment. In fact, loss of accuracy can be related to reduction of somatosensation, decrease of velocity due to paresis and loss of jerkiness to abnormal muscle tone [31]. Before being used in clinical evaluations, these indices have to be validated by comparison with clinical scales [27]. For this reason, we tested the validity of this UL function assessment through instrumental indices by quantifying their ability to distinguish between stroke patients and healthy subjects, by identifying indices that assess different domains of UL movement, and by verifying their correlation with a clinical reference standard. The aim was to give clinicians an objective, quantitative and easy-to-use assessment tool to improve UL function evaluation in neurological diseases.

## Methods

### Participants

Forty-four stroke patients, who were admitted to the Rehabilitation Department of our hospital over a two-year period (2012–2013) were selected for robotic treatment by their referring physicians, and enrolled in the study. Inclusion criteria were: (1) UL hemiparesis due to first unilateral ischemic or hemorrhagic stroke; (2) no serious cognitive deficits; (3) ability to sit (the Trunk Control Test -TCT- item of “balance in sitting position” score ≥12) and be active in a chair for one hour without cardiac, respiratory and/or pain complaints; (4) ability to actively move shoulder and elbow (MI ≥ 14 at both shoulder and elbow [32]) in conditions of load relief. Exclusion criteria were: (1) severe cognitive-perceptual deficits (ataxia, apraxia, heminegligence, receptive aphasia); (2) shoulder pain or subluxation or other neurological/neuromuscular or orthopedic conditions affecting reaching ability; (3) severe ipovisus.

This study is based on a retrospective analysis of data available from the clinical routine in the years 2012–2013. All patients gave informed consent to data treatment in this research study and permission to publish anonymous data and results. The conduction of this retrospective study did not affect patients’ treatment in any way. It was carried out in accordance with the standard ethical principles and was approved by the inner scientific board of our Hospital, which has received the formal approval for the conduction of clinical studies from the local Ethical Committee (Comitato Etico IRST IRCCS e Area Vasta Romagna, CEIIAV).

Normal reference data used in this study came from a sample of 25 healthy right-handed individuals, aged between 21 and 74 years of age (mean age = 46 ± 20 years) and are available in the reference [30]. In this sample of normal subjects, indices were not affected by age.

### Clinical assessments

Residual UL function was assessed for each subject both clinically and instrumentally. UL motor ability was clinically assessed by the WMFT, which refers to the activities domain of the ICF [11], as it provides a quantitative measurement of motor ability through a wide range of functional tasks and evaluates performance time, quality of movement and strength. The WMFT consists of 17 items: two are related to subject strength and the other 15 to subject functional ability. The mean execution time (WMFT-TIME), expressed in seconds, is obtained as the sum of the execution times of each task (with an upper limit of 120 s each) divided by the number of tasks. The total score, also referred to as Functional Ability score (WMFT-FAS), is the sum of the 15 items score (with a 6-point ordinal score from 0 to 5). The maximum total score is 75, with lower scores indicating lower functional levels.

Finally, the TCT item of “balance in sitting position” score was reported in this paper, as well as the MI score at shoulder and elbow, in order to clinically describe patient functional level. Characteristics of participants are shown in Table 1.

**Table 1:** Demographic and clinical characteristics of the sample

Characteristic	*N* = 44
Age, mean ± st.dev. (range)	62 ± 9 (45–79)
Sex, M/F	32/12
Type of disease	
Cerebral ischemia	27
Cerebral hemorrhage	17
Affected side, Left/Right	24/20
Months from lesion, mean ± st.dev. (range)	2.7 ± 3.6 (0.4–19)
WMFT-FAS, mean ± st.dev. (range)	48 ± 16 (10–75)
WMFT-TIME, s, mean ± st.dev. (range)	18.5 ± 24.5 (2.1–81.5)
MI at the shoulder, mean ± st.dev. (range)	21 ± 6 (14–33)
MI at the elbow, mean ± st.dev. (range)	23 ± 5 (14–33)

### Robotic assessment

UL motor ability was instrumentally assessed by a set of numerical indices based on the 3D endpoint trajectory during the Armeo®Spring “Vertical Capture” task, as in reference [30]. The Armeo®Spring device is a UL exoskeleton equipped with seven goniometers and one pressure sensor, which permits free 3D arm movement and provides a support to the weight of the arm, adjustable over five levels. A handle based at the end of the robotic arm, containing a pressure sensor, measures hand grip force. Visual feedback of the subject’s hand position is displayed and used in a set of rehabilitation games and testing exercises. In our study, raw sensor data and the endpoint trajectory were acquired and stored by setting a specific software registry key, as described in the device manual.

Before any acquisition, the physiotherapist adjusted the exoskeleton so that it fit comfortably to the subject’s arm and set the weight support to the minimum level, where the patients were able to maintain their forearm in a horizontal position and to move their UL within a 45-degree range of flexion. The patient, whilst wearing the device, practiced making a few free assisted movements and was then asked to try preforming the “Vertical Capture” task, described below, at the easiest level twice in a row, without worrying about the result and for the purpose of getting used to the device.

The purpose of the “Vertical Capture” task is to assess the functional level of patients. In this task, a target (ladybird) appears on the monitor and the subject has to place the cursor, controlled by the endpoint position, on the target. When a target is hit, it disappears and a new one appears on the screen. Both the number of targets and the area covered by the targets increase with the difficulty level of the exercise, which ranges from 1 to 4.

After practicing for approximately five minutes, each subject executed three repetitions of the “Vertical Capture” task at the maximum difficulty level according to their ability, which was the highest level out of four where the patient reached the highest number of targets (typically 100 %). Thanks to this choice, the data always represented the best performance of each subject, according to their residual ability and can be grouped and compared to the performance assessed by the WMFT. A set of indices was computed based on the 3D endpoint trajectory. These indices are summarized in Table 2 and fully described in reference [30].

**Table 2:** Indices computed based on the 3D endpoint trajectory used for assessing UL movement accuracy, velocity and smoothness

Index, units	Description
Indices of accuracy	
HPR, %	Global Hand Path Ratio, defined as the ratio between the length of the endpoint trajectory during the reaching movement and the minimum distance between the starting point and target, expressed as a percentage. HPR equals 100 % for straight movements and increases with the trajectory curvature and presence of abnormal movements.
locHPR, %	Local Hand Path Ratio, defined as the ratio between the length of the endpoint trajectory and the shortest trajectory within a circle with a 2.5 cm radius centered in the target, expressed as a percentage. The locHPR equals 100 % for straight movements towards the target and increases if the individual reaches the target with multiple adjustments.
vertOS and horOS, cm	Vertical and horizontal overshoot, defined as the excess, if any, in both a vertical and horizontal direction beyond the region delineated by the starting point and target.
Indices of velocity	
maxVel, cm/s	Maximum velocity of the velocity profile of the 3D endpoint trajectory during each single reaching movement.
meanVel, cm/s	Mean velocity of the velocity profile of the 3D endpoint trajectory during each single reaching movement.
meanVel/maxVel, %	Ratio between mean and maximum velocity, expressed as a percentage. This ratio outlines the presence of a movement characterized by brisk movements with stops and starts.
Indices of smoothness	
NVelPeaks, adim	Number of local peaks in the velocity profile. This index counts the number of partial movements used to complete a single reaching movement. Also referred to as number of movement units.
NormJerk, adim	The normalized jerk is a measurement of the trajectory smoothness. NormJerk was computed through numerical differentiation of the 3D endpoint trajectory and a zero phase lag low-pass Butterworth filtering with a cut-off frequency of 10 Hertz. NormJerk tends to be 1 for purely sinusoidal traces and greatly increases in the presence of acceleration variations.

### Statistical analysis

To explore values that have the potential to distinguish between stroke patients and healthy subjects, patient indices were compared to those obtained from healthy, right-handed reference individuals using the non-parametric Mann-Whitney *U* test. As in reference [27], the effect size (ES) was then calculated by dividing the Z-score by the square root of the total number of participants: $ES=Z/\sqrt{N}$.

To verify the discriminant validity of the indices when assessing accuracy, velocity and smoothness in stroke patients UL movement, a factor analysis was performed. Principal Component Analysis (PCA) was applied to the complete dataset to select the most significant features among the set of indices that characterize UL movement. The minimum number of Principal Components (PCs) considered as significant was determined using the Kaiser criterion; i.e. only PCs with an associated eigenvalue greater than one were taken into account. A Varimax rotation was performed to obtain a group of homogeneous and significant variables for each PC [33]. The Kaiser-Meyer-Olkin (KMO) measure was used to assess the adequacy of the analysis [34].

Then a correlation analysis was used to verify the concurrent validity of the indices with a recognized clinical reference standard. Correlations between the investigated parameters and both the WMFT-FAS and WMFT-TIME were analyzed by the Spearman’s rank correlation coefficient. The strength of the correlation coefficients was interpreted according to Guilford [35]: 0.0–0.2 little if any; 0.2–0.4 weak; 0.4–0.7 moderate; 0.7–1.0 strong. As a result of having a sample size of 44 subjects, we will be able to correctly reject the null hypothesis of no correlation with a power of 66 % in the case of medium effect size (i.e. |rho| = 0.3) and with a power of at least 98 % in the case of a large effect size (i.e. |rho| > =0.5). The power analysis was conducted in G-POWER using an alpha of 0.05 and a sample size of 44.

These analyses allowed for the selection of the most appropriate indices to be used in the analysis of UL motion in stroke patients among the set of available indices, based on their sensitivity to the pathological condition, their ability to assess specific domains of UL movement and their concurrent validity with the clinical reference standard.

Statistical analysis was performed using SPSS statistical software (version 21.0, SPSS Inc., Chicago, IL, USA). Statistical significance was set at 5 % for all analyses.

## Results

### Ability of indices to distinguish between stroke patients and healthy subjects

The mean values of indices in the sample are presented in Table 3, along with the corresponding normal reference values. As expected, statistically significant differences between patient values and normal values were found.

**Table 3:** Values of indices for stroke patients and healthy subjects. The 10^th^-90^th^ percentile ranges are indicated

Indices	Healthy subjects	Stroke subjects	|Effect size|
HPR, %	121–154	136–273 ^†^	0.58
horOS, cm	0.8–2.2	1.1–3.6 ^†^	0.35
vertOS, cm	0.6–1.7	0.3–1.8	-
locHPR, %	129–231	132–371	-
meanVel, cm/s	1.8–3.5	1.0–2.8 ^**^	0.55
maxVel, cm/s	5.1–10.0	3.1–10.1 ^*^	0.42
meanVel/maxVel, %	34.4–40.1	27.6–37.2 ^**^	0.52
NVelPeaks, adim	1.3–1.8	1.4–4.7 ^†^	0.74
NormJerk, adim	72–356	227–24 823 ^†^	0.63

Values of instrumental indices used to assess upper limb reaching motion (10^th^ – 90^th^ percentile range) in healthy subjects (*N* = 25) and the sample of stroke patients enrolled in this study (*N* = 44). Statistical comparison was assessed by the non-parametric Mann-Whitney *U* test. Effect size is reported for significant differences. Legend: ^*^ *p* < 0.05; ^**^ *p* < 0.01; ^†^ *p* < 0.001

ES ranged between 0.35 and 0.74 and was higher for indices assessing lack of smoothness in movement trajectory. Two specific indices, number of peaks in the velocity profiles (NVelPeaks) and Normalized Jerk (NormJerk), showed the best ability to distinguish between normal and pathological subjects. In general, according to Cohen’s ES interpretation rule, a discriminant ability of the pathological condition ranging from medium to large was reached by at least one index for all of the three domains investigated. Based on ES, the global Hand Path Ratio (HPR) should be selected to assess accuracy, mean velocity (meanVel) should be selected to assess velocity and NVelPeaks to assess the lack of trajectory smoothness.

### Discriminant validity of indices

**Fig. 1:**
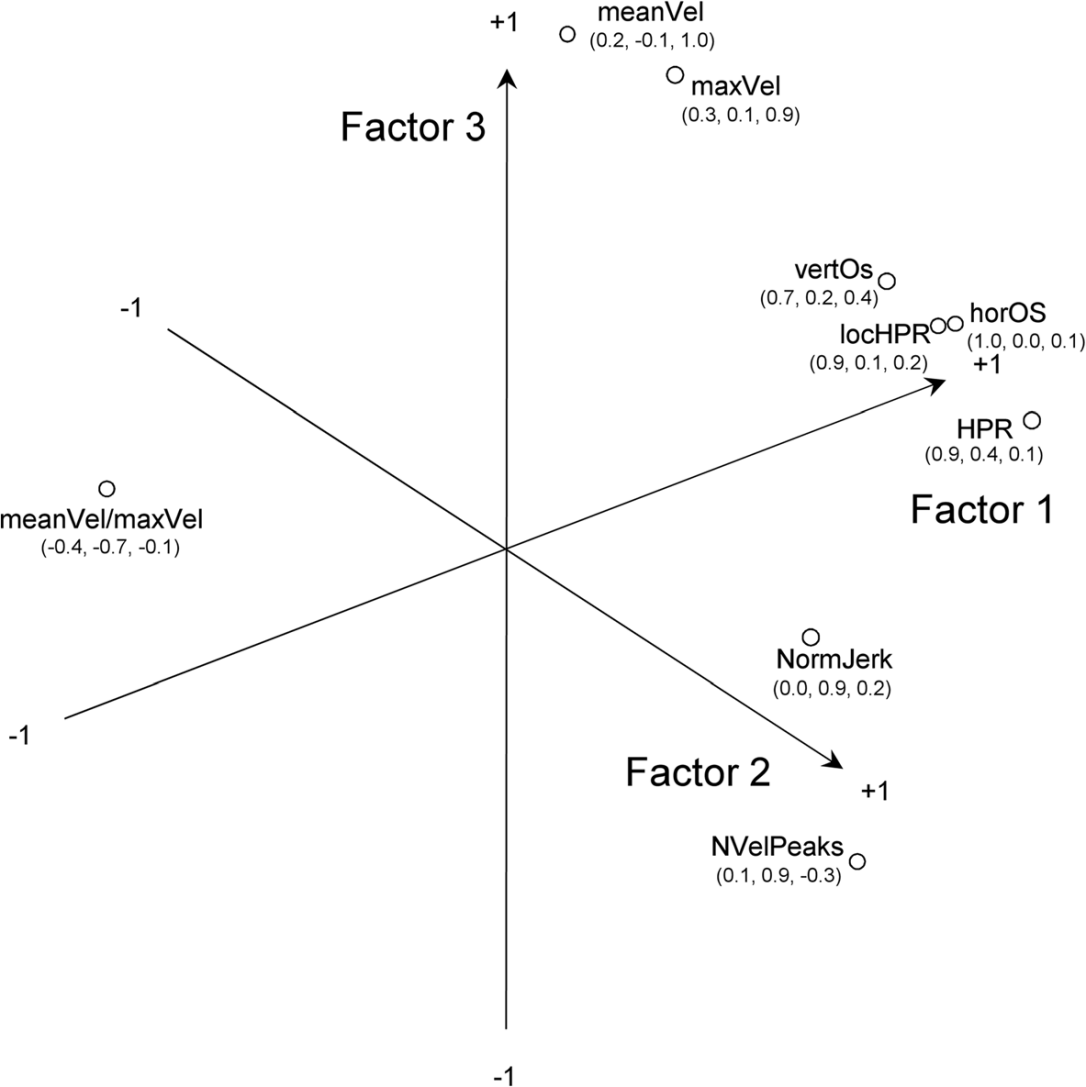
Graphic representation of factor analysis results. All indices except meanVel/maxVel resulted in being grouped together within a specific factor. Factor 1 deals with accuracy of movement trajectory, Factor 2 deals with trajectory smoothness and Factor 3 deals with velocity

Factor analysis led to the identification of three significant factors, which accounted for 87 % of the variance in the entire set of parameters. KMO was 0.707, thus confirming a good factor analysis. Figure 1 presents the output of the rotation procedure. All indices expected to assess movement accuracy (see Table 2) resulted grouped together in factor 1, both indices assessing movement smoothness were grouped in factor 2 and two of the indices assessing velocity, meanVel and maximum velocity (maxVel), were grouped in factor 3. For these indices, their discriminant ability to assess a specific domain of UL movement (e.g. smoothness) was confirmed. The variable ratio between mean and maximum velocity (meanVel/maxVel) did not clearly fall within a single domain and was, therefore, excluded from further analysis.

### Concurrent validity of indices

Correlation analysis was used to verify the concurrent validity of the proposed indices by comparing them to a clinical scale, the WMFT, which was used as a reference standard. Weak to moderate significant correlations were found for a subset of the indices, as presented in Table 4.

**Table 4:** Spearman’s *rho* correlation coefficient and statistical significance (P Value) for both the WMFT Functional Ability Score (WMFT-FAS) and the mean execution time (WMFT-TIME) in relation to nine instrumental indices that assess trajectory accuracy, velocity and smoothness during the execution of Armeo®Spring-assisted upper limb reaching tasks

	WMFT-FAS		WMFT-TIME	
Indices	*rho*	*P* value	*rho*	*P* value
HPR, %	**−0.36**	**0.018**	**0.44**	**0.003**
horOs, cm	−0.10	0.524	0.10	0.501
verOs, cm	0.036	0.815	0.07	0.659
locHPR, %	−0.22	0.145	0.23	0.132
meanVel, cm/s	**0.31**	**0.039**	−0.26	0.082
maxVel, cm/s	0.15	0.328	−0.10	0.514
meanVel/maxVel, %	**0.45**	**0.002**	**−0.44**	**0.003**
NVelPeaks, adim	**−0.50**	**0.001**	**0.56**	**<0.001**
NormJerk, adim	**−0.50**	**0.001**	**0.55**	**<0.001**

Bold font is used to highlight statistically significant correlations (*P* < 0.05). Within these results, the lack of correlation between WMFT and the local accuracy index locHPR might be affected by a 2nd-type error

The correlation coefficients between the indices and the WMFT-FAS and WMFT-TIME have similar absolute values and opposite signs. This result is due to the fact that the WMFT-TIME decreases with patient improvement, whilst the WMFT-FAS increases with patient improvement. The lower the WMFT-FAS was, the lower were the accuracy and/or velocity and/or smoothness of the movements. Similarly, the longer the WMFT-TIME was, the lower were accuracy, velocity and/or smoothness.

Among the indices that describe movement accuracy, HPR, which is related to overall accuracy during the gesture, was correlated with both the WMFT-FAS and the WMFT-TIME, while the local accuracy index and the overshooting indices (Table 2) did not. Consequently, the HPR can be used to provide a quantitative assessment of an individual’s UL movement accuracy. Among indices based on endpoint velocity, meanVel was correlated with the WMFT-FAS and almost significantly correlated (*p* = 0.082) with the WMFT-TIME. Therefore, in stroke patients, meanVel can be used to provide a quantitative assessment of an individual’s overall UL speed. Indices assessing trajectory smoothness presented a strongly significant correlation (*p* < 0.001) with both the WMFT-FAS and the WMFT-TIME and can be used to provide a quantitative assessment of an individual’s UL movement smoothness.

### Construct validity of indices

Table 5 summarizes the performance of the indices in terms of the ES in the comparison between stroke patients and healthy subjects, the discriminant validity and the concurrent validity. Based on Table 5, we selected a single index for each of the assessed movement domains, which are HPR for accuracy, meanVel for velocity and NVelPeaks for smoothness. We consider that the number of peaks in the velocity profile, i.e. the number of movement units within a single reaching task, is preferable to the NormJerk variable because of its ease of computation and sharing with clinicians.

**Table 5:** Summary of the results that allowed for the definition of the construct validity of the UL assessment carried out in this study

Index, units	Effect size ≥ 0.5 stroke patients vs. normal subjects	Discriminant validity of movement domains	Concurrent validity with the WMFT	Construct validity
Indices of accuracy				
HPR, %	Yes	Yes	Yes	Yes
locHPR, %	No	Yes	No	
vertOS and horOS, cm	No	Yes	No	
Indices of velocity				
meanVel, cm/s	Yes	Yes	Yes	Yes
maxVel, cm/s	No	Yes	No	
meanVel/maxVel, %	Yes	No	Yes	
Indices of smoothness				
NVelPeaks, adim	Yes	Yes	Yes	Yes
NormJerk, adim	Yes	Yes	Yes	Yes

For each movement domain, at least one index satisfied all the requirements for construct validity

### Indices added value to clinical routine

**Fig. 2:**
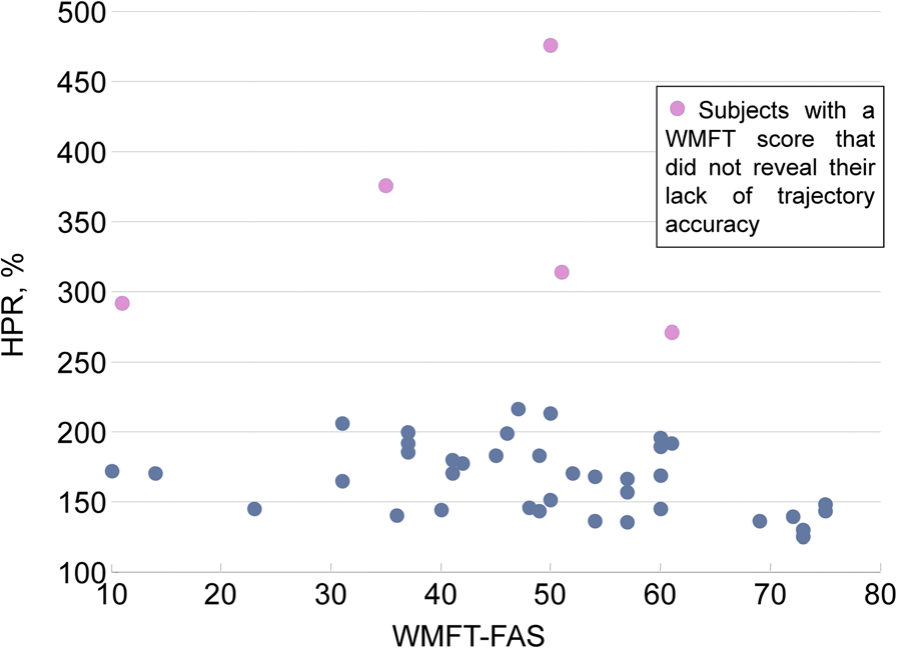
Scatterplot of HPR versus WMFT-FAS. In general, HPR decreases when WMFT-FAS increases. For a few subjects however, highlighted in the figure, clinical assessment by the WMFT-FAS did not reveal a lack of control in movement accuracy. Thanks to the weight support, even the most compromised patients in the sample were able to complete the task at the easiest level with a success rate ranging from 80 to 100 %

**Fig. 3:**
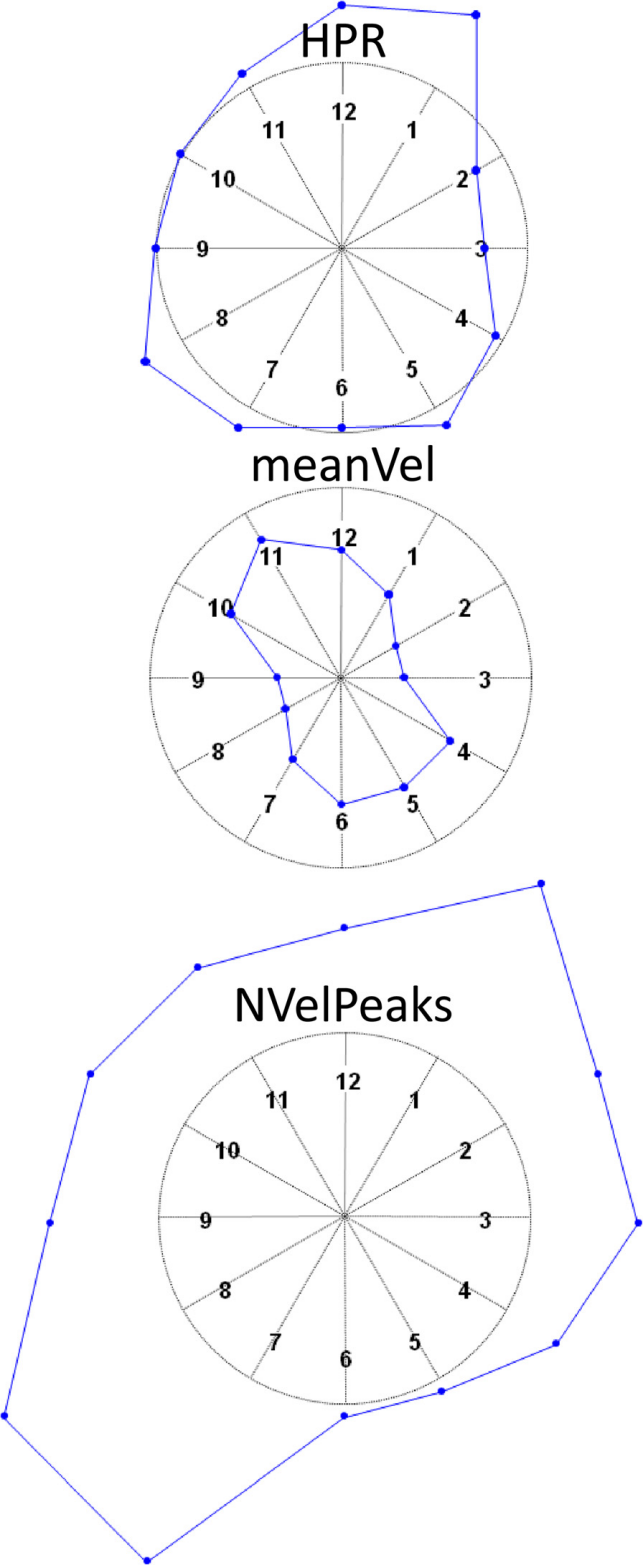
Graphic representation of computed indices for one patient in the sample, based on the “Vertical Capture” task of the Armeo®Spring device being repeated three consecutive times. Similarly to a clock, the numbers indicate movements in a specific direction (e.g. 1–7, or 3–9). Indices were grouped based on the direction of the movement and normalized to the normal reference value for that direction. The median value was then computed and plotted. Due to the normalization procedure, values close to the normal reference are close to the circumference; values greater than normal are outside the circumference (as per HPR) and values lower than normal are inside the circumference (as per meanVel). This representation provides a glimpse of one patient’s UL movement impairment. This subject displayed jerky movements with reduced velocity but displayed sufficient control of the movement trajectory. Directions 1–7 and 2–8 appear to be the most compromised. A better overall accuracy (HPR) was achieved in the horizontal movements (direction 3–9), possibly due to a greater control ability, despite jerkiness (NVelPeaks), which resulted in a lower velocity (meanVel)

The added value to clinical assessment provided by the indices can be seen in Figs. 2 and 3. Figure 2 presents the scatterplot of the HPR versus WMFT-FAS. A coarse linear trend can be appreciated. Interestingly, some patient data are considerably distant from this trend; i.e. patients with equal WMFT-FAS may have significantly different HPR values.

A graphic representation of the indices for one of the sample patients is presented in Fig. 3. In this plot, indices were computed for each single reaching movement, grouped based on the movement direction and normalized with respect to normal reference values. Consequently, normal values are located on the circumference, values below normal are located inside the circumference and values above normal are located outside the circumference. Figure 3 is useful to identify the directions for which UL movement is compromised. Directions 1–7 and 2–8 are those with the worst indices for this patient.

## Discussions

In this study, we investigated the validity of a set of instrumental indices, which were computed based on the endpoint trajectory of the Armeo®Spring device and previously described in literature [30], in order to assess UL movement performance in sub-acute stroke patients. In more detail, we analyzed the following three aspects: the ability of the indices to discriminate between stroke patients and healthy subjects; the discriminant validity of the indices in assessing different movement domains; the concurrent validity of the indices with the WMFT, which is a clinical reference scale for assessment of residual UL function. The presence of all three above-mentioned characteristics was used to state the construct validity of the indices. The main result of this study is the selection and validation of three indices that assess residual UL motor ability in sub-acute stroke patients in terms of accuracy, velocity and smoothness.

Our results confirmed the validity of the Hand Path Ratio in the assessment of the overall difficulty of motor execution and control [29]. In our study, HPR was nearly 40 % higher in affected patients than in healthy individuals (*p* < 0.001) and showed a good performance when discriminating between stroke patients and normal subjects (|ES = 0.58|), in line with previous studies by Coderre et al. [36] and Otaka et al. [27]. The HPR showed a moderate correlation with the WMFT-FAS and the WMFT-TIME (rho ~ 0.4) and, from the factor analysis, it resulted as belonging to the accuracy domain with minor influence from the lack of smoothness domain. This result is similar to available literature, in which measurements of movement accuracy based on the endpoint trajectory resulted correlated with the severity of clinical symptoms in stroke patients, as summarized in the systematic review by Nordin et al. [29]. Along with these similarities, the method described in this study to assess residual UL ability in stroke patients offers two improvements with respect to literature. Firstly, the use of an adjustable weight support permits the assessment of compromised patients right from the early, sub-acute phase of their disease. Secondly, the selected task allows for identifying the most compromised movement directions, as shown in Fig. 3, an objective that was also suggested in the conclusion of the review by Nordin et al. [29] and in accordance with previous conclusions from Colombo, Micera et al. [37].

Validity was not verified for the indices assessing local accuracy. Only a few subjects presented difficulty in keeping the cursor within the rectangle delineated by the starting and ending points, as assessed by horizontal overshooting (horOS) and vertical overshooting (verOS). It is reasonable to suppose that overshoots were comparable to those of normal subjects due to the gravitational support provided by the device. Likewise, the lack of control in the target area, assessed by local HPR (locHPR), did not differentiate stroke patients from healthy subjects and did not significantly correlate with the clinical reference standard. Finally, the lack of concurrent validity with both the WMFT-FAS and the WMFT-TIME (Table 4) does not allow for further use of these indices dealing with local accuracy in the assessment of stroke patients. 

Mean velocity was significantly reduced in our sample when compared to the normal reference with a medium ES, as per Cohen’s classification. It showed a moderate correlation with the WMFT-FAS and the WMFT-TIME and, from the factor analysis, it fell markedly within the velocity domain with negligible contributions from other domains. The validity of meanVel in assessing UL motor impairment in stroke patients was proven by these results, which correspond to findings in literature, where the mean endpoint velocity during reaching movements was found to distinguish between acute and chronic stroke patients [29] and to be related to the FMA score in acute patients [28, 37].

In our study, the maximum velocity did not correlate with clinical indicators of UL ability. This result could be explained by the fact that the WMFT evaluates the overall performance of patients, thus correlating to the mean velocity, rather than the ability of completing a task as fast as possible. In literature that deals with exoskeletons and robotic devices, peak velocity was found to correlate with clinical scores in a few studies on the MIT-Manus [28, 38]. However, this device calls for planar movements of the hand, hence differing from the 3D movements required by the “Vertical Capture” task of the Armeo®Spring device. In our study, the mean velocity discriminated stroke patients from healthy subjects better than peak velocity. This distinction is similar to the one we found between HPR and locHPR. Indeed, meanVel is a global indicator that depends on the execution of the entire reaching movement, whilst peak velocity is a local indicator that does not take into account for any other characteristics of the reaching movement. An interesting comparison can be made with a study by Edwards et al. [14]. They investigated a sample of sub-acute stroke patients with UL motor performances similar to those of our sample (recruitment at 42 ± 40 days after lesion) by assessing both the WMFT and a set of kinematic variables computed from motion capture data. Interestingly, the wrist peak velocity measured during a reaching task was much higher (62 ± 35 cm/s) compared to the one obtained in our sample. In contrast, during a “reach to grasp” task of a small cylinder peak velocity was lower (11 ± 9 cm/s) and similar to the one we obtained in this study. The former difference could be accounted for because, in our study, the subjects were not able to lean their trunk towards the target during the assisted task, since the UL was fitted to the exoskeleton. Leaning the trunk forward is the main compensatory mechanism used by stroke patients with impaired elbow extension when reaching for a target and results in higher endpoint velocity [39]. The latter similarity could be accounted for by the need for similar control at the end of the reaching movement. This might result in similar low peak velocities during the task. The control of trunk compensatory mechanisms made possible by the exoskeleton used in this study is a further improvement in the instrumental assessment of UL function, as recommended in the review by Nordin and colleagues [29].

The presence of jerky movements was assessed by both smoothness indices used in this study (NVelPeak and NormJerk). The number of velocity peaks during reaching movements reflects the presence of “sub-movements” [28, 38], or “movement units” [40, 41], and tends to decrease when smoothness improves [42]. In our study on 3D movements of the UL, these two indices showed a high ability in discriminating between patients and healthy individuals (|ES| = 0.74 and |ES| = 0.63) and a statistically significant (*p* < 0.001) medium to large correlation (rho = 0.50–0.56) with the clinical reference standard, showing the best performance in describing the ability of stroke patients across the entire set of indices. Factor analysis confirmed that the NVelPeak and NormJerk fell within the specific domain of lack of smoothness, distinctly from movement accuracy and velocity. These results appear to be in accordance with the physiopathology of motor impairment in subjects who have suffered a stroke, which is characterized by impaired recruitment and de-recruitment, exaggerated reflexes to fast stretches (spasticity), muscle shortening coupled by increased stiffness, abnormal muscle patterns and co-contractions, resulting in movement fragmentation [43]. In literature, the number of peaks in the speed profile has been used to quantify trajectory smoothness in reaching tasks performed by stroke patients in the 2D [28] and 3D-Space [14, 44] and was found to correlate with the clinical stage of motor recovery, in accordance with our results. Similarly, the number of peaks in the hand velocity profile obtained by Colombo et al. [45] and by Otaka et al. [27] was significantly correlated with clinical scales, including the WMFT-FAS. Interestingly, these results were obtained with various robotic devices and exoskeletons, consequently suggesting that the number of peaks in the endpoint velocity profile, during reaching tasks, can be used as an assessment tool for smoothness impairment regardless of the type of robotic device used.

While the majority of published results deals with chronic stroke patients, the robotic assessment described in this study is based on the validation of indices in a sample of patients in the sub-acute stage of the disease, which is when most rehabilitation is carried out, and when the need for UL performance assessment is greatest. The focus on early-stage stroke patients of this study goes beyond what is present in current literature, where validation of clinical indices was often investigated in chronic patients [27, 38, 46]. Chronic patients are generally clinically stable and easier to enroll, but present a low degree of potential recovery. Assessment of sub-acute in-patients was made possible by the UL weight support of the chosen device, which allowed for even very weak patients to be tested at the start of their rehabilitation. The inclusion of highly compromised in-patients also goes beyond what is present in current literature. As measured by the MI (see Table 1), the paresis level ranged from moderate to normal (i.e. MI = 14 and MI = 33 at elbow and/or shoulder respectively), thus covering a very wide range of motor impairments and giving external validity to our results. In this approach, besides identifying the most compromised directional movements, it becomes possible to track longitudinal changes in the motor recovery of stroke patients by repeating instrumental measurements throughout the rehabilitation period.

The selection of the WMFT as the reference standard, as in reference [14], was appropriate for the validation purpose of our study. In fact, the WMFT scale assesses overall UL limitations, according to the ICF activity domain [10, 11]. In contrast, other clinical scales for UL evaluation used in studies on robotic indices, such as the FMA and the Brunnstrom Recovery Stage, focus only on motor impairment that is related to the body function and structure ICF domain. Moreover, the “Vertical Capture” task chosen for this study allows for the assessment of 3D reaching movements, which are essential for many everyday activities [27] and, therefore, preferable to the 2D movements called for by planar robots and closer to the WMFT setting for clinical assessment of function. As the indices are computed from data acquired during the “Vertical Capture” task with the Armeo®Spring device, it is essential that this specific task be used only to assess UL function and not as a training task throughout the rehabilitation period.

We consider these indices to offer a fast and useful support in the UL rehabilitation of stroke patients, as they can be obtained just prior to commencing a rehabilitation session, by the same operator, in the same place and without any special preparation. Moreover, instrumental indices can be repeated many times to obtain a mean value which can prove more robust than a single score obtained by clinical scales [26]. Finally, the indices were able to detect differences in accuracy, velocity or smoothness in patients with the same WMFT-FAS and WMFT-TIME, showing greater sensitivity than clinical scales in measuring motor recovery in stroke patients, in accordance with literature [26]. Thus, indices can be used both to improve the decision making in UL rehabilitation and to assess the effectiveness of the delivered treatment.

This study had some limitations. We did not control the positions in which the targets appeared on the screen; these were randomly generated by the device’s software. As a result, identifying the most compromised directional movement can be inaccurate if a patient can complete the easiest task only (level 1), with no more than randomly generated 12 targets. The acquisition of three consecutive trials at the maximum difficulty level according to individual patient’s ability should have minimized this possible error.

From a methodological point of view, a repeatability study between two sessions should be carried out in order to determine the minimum significant variation of the selected indices [30], for patients with the same inclusion/exclusion criteria. A longitudinal study should also be carried out to evaluate the responsiveness and expected change of the selected indices in relation to the clinical improvement in the UL motor ability of stroke patients. Finally, future studies could validate the use of this set of indices with other neurological conditions.

## Conclusions

Construct validity has been proven for three of the investigated indices: the hand path ratio, the mean velocity and the number of peaks in the velocity profile, which assess movement accuracy, velocity and smoothness, respectively, and can be used as tools to integrate clinical evaluation of UL function in stroke patients from the sub-acute recovery stage.

## Abbreviations

ADL, activities of daily life; ES, effect size; FMA, Fugl-Meyer Motor Performance Assessment; horOS, horizontal overshooting; HPR, global Hand Path Ratio; ICF, International Classification of Function, Disability and Health; KMO, Kaiser-Meyer-Olkin; locHPR, local HPR; maxVel, maximum velocity; meanVel/maxVel, ratio between mean and maximum velocity; meanVel, mean velocity; MI, Motricity Index; NormJerk, Normalized Jerk; NVelPeaks, number of peaks in the velocity profiles; PCA, Principal Component Analysis; PCs, Principal Components; TCT, trunk control test; UL, upper limb; verOS, vertical overshooting; WMFT, Wolf Motor Function Test
